# Structural Determinants of Substrate Specificity of SplF Protease from *Staphylococcus aureus*

**DOI:** 10.3390/ijms22042220

**Published:** 2021-02-23

**Authors:** Natalia Stach, Abdulkarim Karim, Przemyslaw Golik, Radoslaw Kitel, Katarzyna Pustelny, Natalia Gruba, Katarzyna Groborz, Urszula Jankowska, Sylwia Kedracka-Krok, Benedykt Wladyka, Marcin Drag, Adam Lesner, Grzegorz Dubin

**Affiliations:** 1 Malopolska Center of Biotechnology, Jagiellonian University, 7a Gronostajowa St., 30-387 Krakow, Poland; natalia.stach@uj.edu.pl (N.S.); abdulkarim.karim@su.edu.krd (A.K.); kpustelny@interia.pl (K.P.); urszula.jankowska@uj.edu.pl (U.J.); 2 Department of Biology, College of Science, Salahaddin University-Erbil, Kirkuk Road, 44002 Erbil, Kurdistan Region, Iraq; 3 Faculty of Chemistry, Jagiellonian University, 2 Gronostajowa St., 30-387 Krakow, Poland; przemekgolik87@gmail.com (P.G.); radoslaw.kitel@uj.edu.pl (R.K.); 4 Faculty of Chemistry, University of Gdansk, 63 Wita Stwosza St., 80-308 Gdansk, Poland; natalia.gruba@ug.edu.pl (N.G.); adam.lesner@ug.edu.pl (A.L.); 5 Department of Chemical Biology and Bioimaging, Wroclaw University of Science and Technology, 27 Wyb. Wyspianskiego St., 50-370 Wroclaw, Poland; katarzyna.groborz@pwr.wroc.pl (K.G.); marcin.drag@pwr.edu.pl (M.D.); 6 Department of Physical Biochemistry, Faculty of Biochemistry Biophysics and Biotechnology, Jagiellonian University, 7 Gronostajowa St., 30-387 Krakow, Poland; sylwia.kedracka-krok@uj.edu.pl; 7 Department of Analytical Biochemistry, Faculty of Biochemistry Biophysics and Biotechnology, Jagiellonian University, 7 Gronostajowa St., 30-387 Krakow, Poland; benedykt.wladyka@uj.edu.pl

**Keywords:** serine protease, substrate specificity, crystal structure, virulence factor, *Staphylococcus aureus*

## Abstract

Accumulating evidence suggests that six proteases encoded in the *spl* operon of a dangerous human pathogen, *Staphylococcus aureus*, may play a role in virulence. Interestingly, SplA, B, D, and E have complementary substrate specificities while SplF remains to be characterized in this regard. Here, we describe the prerequisites of a heterologous expression system for active SplF protease and characterize the enzyme in terms of substrate specificity and its structural determinants. Substrate specificity of SplF is comprehensively profiled using combinatorial libraries of peptide substrates demonstrating strict preference for long aliphatic sidechains at the P1 subsite and significant selectivity for aromatic residues at P3. The crystal structure of SplF was provided at 1.7 Å resolution to define the structural basis of substrate specificity of SplF. The obtained results were compared and contrasted with the characteristics of other Spl proteases determined to date to conclude that the *spl* operon encodes a unique extracellular proteolytic system.

## 1. Introduction

*Staphylococcus aureus* asymptomatically colonizes 20–50% of the human population, inhabiting mainly the mucosa of the nasal cavity and throat [1]. At the same time, the bacterium is a dangerous human pathogen, a major cause of nosocomial and community-acquired infections. The severity of staphylococcal infections varies from relatively harmless skin conditions (impetigo, boils, folliculitis, abscesses), through to food poisoning and life-threatening events including endocarditis, toxic epidermal necrolysis, meningitis, toxic shock syndrome, pneumonia, osteomyelitis, and sepsis [2]. The steadily increasing resistance of *S. aureus* to currently available antibiotics poses a significant threat to the health care system.

The complexity in the balance of commensal and virulent phenotypes in staphylococci is still insufficiently understood. What is well established, is that in laboratory conditions, the extracellular proteome changes significantly along with the growth phase. In the early growth phase, expression of surface proteins, primarily adhesive factors, predominates. At later phases, surface protein synthesis is suppressed, and replaced by secretion of soluble factors including proteases that “shave” the cell off the remaining surface proteins. The transition is controlled by global regulatory systems of gene expression, primarily *agr* (accessory gene regulator) and *sar* (staphylococcal accessory regulator) [3,4]. *Sar* promotes the production of adhesins while *agr* controls the expression of migration related factors, and thus systems switching is believed to reflect the transition from the early colonization state of the disease to the late invasive state, although the evidence is largely indirect [5].

In the late exponential growth phase, staphylococci start to secrete three catalytic classes of proteases including serine-, cysteine-, and metallo-enzymes [6]. Several staphylococcal proteases were implicated in virulence and thoroughly characterized in terms of their enzymatic activity, but the group of six *spl* operon encoded enzymes still largely eludes understanding in these contexts. The discovery of the first *Spl* protease was reported in 1997 by Rieneck and collaborators, who examined the reactivity of human serum, obtained from patients with staphylococcal endocarditis, to identify SplC as a single protein eliciting a strong humoral response. These results suggest SplC expression during infection [7]. The entire *spl* operon was described a few years later, and demonstrated to locate on a pathogenicity island, and to encode from a single up to six homologous serine proteases (SplA-SplF) in different strains [8]. Like other staphylococcal proteases, the expression of *spl* operon is induced in the late exponential growth phase, being regulated by *agr* [9]. Mechanisms by which Spls influence staphylococcal virulence only start to be uncovered. Spls were shown to be required for *S. aureus* to cause disseminated lung damage during pneumonia. SplA was demonstrated to cleave mucin A, which was suggested to be the possible mechanism of bacterial spread in the lungs [10]. In another study, Stentzel and collaborators suggested the role of Spl proteases as triggering allergens in human asthma, and demonstrated that SplD inhalation induced lung inflammation in mice [11]. Teufelberger and colleagues studied the related pathways to show that intratracheal exposure to SplD led to Il-33 production and resulting eosinophilia [12]. Despite these early findings, the mechanisms by which Spl proteases are implicated in staphylococcal virulence require further elucidation.

We have earlier characterized enzymatic activity of five out of the six proteases encoded within the *spl* operon. Interestingly, Spl proteases demonstrated complementary specificities at the P1 subsite, but also a stricter preference extending beyond this classical specificity determinant within the S1 (chymotrypsin) family. SplA preferentially recognized a consensus of (W/Y)-L-Y*(T/S) (asterisk indicates the cleavage site) [13], SplB preferred substrates containing W-E-L-Q* sequence [14], and SplD recognized R-(Y/W)-(P/L)-(T/L/I/V)*S consensus [15], while SplE that of (L/V)-(W/Y/F)-L-(H/Q)*(S/G) [16]. The structural basis guiding the substrate specificities in all the above enzymes were explained by structural studies. SplC showed no activity against any of the numerous tested peptides and protein substrates, and was characterized by a deformed active site in x-ray crystallography. It was proposed, however, that a specific, yet unidentified substrate, could activate the enzyme to release proteolytic activity [17]. Currently, SplF remains the last Spl protease, for which the substrate specificity has not been determined, and it is most exciting to examine whether it complements the remaining enzymes, creating a complex proteolytic system, and if so, the structural basis of such specificity.

To complete the biochemical characterization of proteases of the *spl* operon, in this study, we describe the enzymatic and structural characteristics of SplF as well as compare and contrast them with currently available characteristics of remaining proteases of the *spl* operon. The substrate specificity of SplF is determined using combinatorial libraries of fluorogenic substrates. The molecular mechanism determining the substrate preference is explained using x-ray crystallography. Finally, we discuss the mechanism of precise N-terminal trimming in controlling the activity of the SplF protease.

## 2. Results

### 2.1. Heterologous Expression System for Production of SplF Protease

The recombinant SplF protease variants were obtained by heterologous expression in *E. coli*. His_6_-SplF and Met-Gly-SplF-His_6_ were expressed from pETDuet-1. SplF-His_6_ was expressed from pET20b as a secreted protein to ensure appropriate N-terminal processing, and purified from the culture medium. In all cases, the yield was approximately 1–1.5 mg of purified protein from 1 L starting bacterial culture. All SplF variants were proteolytically active when tested in zymography with β-casein as a substrate.

An inactive mutant of the protease (His39Ala) was used for crystallization. A GST (glutathione S transferase) LVPRGS SplF(His39Ala) fusion was expressed from pGEX-5T plasmid [18], and the tag was removed with thrombin, yielding Gly-Ser SplF(His39Ala). Approximately 5 mg of purified protein was obtained per 1 L of the starting bacterial culture. Interestingly, wild type SplF did not express from this plasmid, likely due to residual toxicity.

### 2.2. SplF Selects Methionine at P1 Subsite

**Figure 1:**
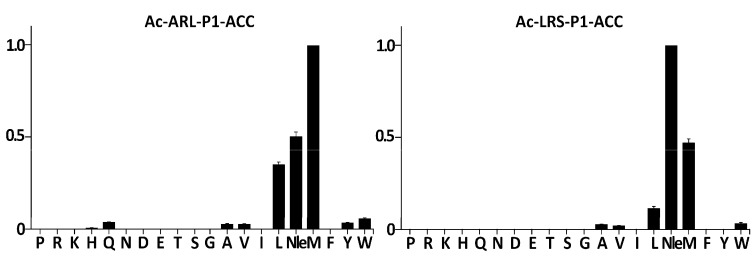
Substrate specificity of SplF protease at the P1 subsite. Substrate preference of SplF at the P1 subsite was determined using two fluorogenic substrate libraries: Ac-Ala-Arg-Leu-P1-ACC and Ac-Leu-Arg-Ser-P1-ACC. Vertical bars indicate the activity of the enzyme against each tested sublibrary normalized to the most active sublibrary. Residues fixed at the P1 subsite are indicated with the single-letter amino acid code.

The specificity of serine proteases belonging to the S1 family is usually determined at the P1 subsite [19], that is, by the residue which carboxyl group is released upon peptide bond hydrolysis. Therefore, the specificity of SplF was initially tested using two combinatorial libraries of fluorogenic substrates of the following structures: Ac-Ala-Arg-Leu-P1-ACC and Ac-Leu-Arg-Ser-P1-ACC, originally designed to assay the specificity of cathepsin L [20] and caspases [21], respectively. Each of these libraries contained 20 sub-libraries, each of which contained a particular proteinogenic amino-acid or norleucine (Nle) at the P1 position. Nle was included as a non-oxidizable analog of oxidation-prone Met. All libraries contained the same leaving fluorescent group at the P1′ position (ACC). When the Ac-Ala-Arg-Leu-P1-ACC library was contacted with purified SplF, only substrates containing Met, Nle, and Leu were significantly hydrolyzed (Figure 1, left panel). In the case of the Ac-Leu-Arg-Ser-P1-ACC library, an identical P1 preference toward long aliphatic sidechains was detected save that the Nle containing substrate was now preferred over the Met containing substrate (Figure 1, right panel), demonstrating a certain influence of further substrate positions at the P1 preference. Significantly, the majority of the remaining residues were excluded at the P1 position while aromatic (Trp, Tyr), β-branched (Ala, Val), and Gln residues were accepted with low efficiency only.

### 2.3. The Substrate Preference of SplF Extends beyond the P1 Subsite

**Figure 2:**
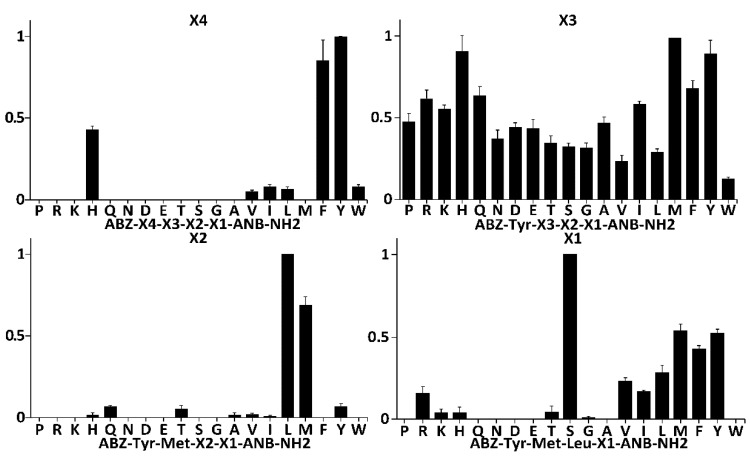
Substrate specificity of SplF determined using combinatorial libraries of synthetic tetrapeptide substrates (LSTS). A library of a general structure Abz-X4-X3-X2-X1-Anb containing 19 sublibraries each having a defined proteinogenic amino acid at the X4 position (X-axis) and an equimolar mixture of these residues at other positions was contacted with SplF and the release of fluorescence was monitored (Y-axis, normalized to the most active sublibrary). X4 position was fixed with the most active residue and X3 scanning was performed in a similar manner. The procedure was repeated until all positions of the library were deconvoluted, yielding a consensus substrate Abz-Tyr-Met-Leu-Ser-Anb.

To probe the specificity of SplF at subsites other than P1, and to select the most kinetically preferred substrate, libraries of synthetic tetrapeptide substrates (LSTS) were deconvoluted with the protease (Figure 2 and Figure S1) [22]. To this end, fluorescence quenched substrates of a general structure Abz-X4-X3-X2-X1-Anb were contacted with the protease while monitoring Abz fluorescence. The first library contained 19 sub-libraries, each of which contained particular proteinogenic amino acid at the X4 position and equimolar mixture of those amino acids at remaining positions. Diversity of sub-libraries at the X3, X2, and X1 positions was monitored by LC-MS. Within such libraries, SplF selected only substrates with bulky aromatic sidechains of Tyr, Phe, and His, but not tryptophan (Figure 2, panel X4). In the next library, the X4 position was fixed with the most preferred residue from the prior selection cycle (Tyr), while the library contained 19 sublibraries, each of which had a specific proteinogenic amino acid at the X3 position. No significant preference was seen using such designed substrates (Figure 2, panel X3), although certain propensity toward substrates with bulky sidechains (save Trp) could have been distinguished. The X3 was fixed with the most efficiently recognized residue (Met), and the X2 position was deconvoluted in a manner similar to X4 and X3. SplF efficiently selected only substrates containing leucine, and methionine at X2. After fixing X2 with the most preferred residue (Leu), SplF demonstrated significant preference for serine at X1, but also recognized hydrophobic sidechains save for tryptophan.

The site of hydrolysis within the preferred substrate selected by full deconvolution of the library (Abz-Tyr-Met-Leu-Ser-Anb) was not immediately obvious given that Met-Leu and Leu-Ser peptide bonds satisfied the P1 specificity of SplF determined earlier in this study. Therefore, the consensus substrate and three variants containing Met, Tyr, and Lys at the X1 subsite were resynthesized and the cleavage site was established using LC-MS exclusively at the Leu-X1 peptide bond in all four substrates tested. No indication of hydrolysis of the Met-Leu peptide bond was obtained. Such results suggest that SplF specificity is determined not only at P1, but also at further, nonprimed subsites, while the primed subsites have little influence on substrate selection.

Fluorescence is released from a quenched substrate after hydrolysis of any peptide bond within its sequence, and thus the Xn sites do not necessarily correspond to Pn sites in Schechter and Berger notation. In our experiment, X1 and X2 library selections clearly corresponded to the P1’ and P1 subsites, respectively, as evidenced by the hydrolysis site in the final selected substrate. However, it was not immediately clear if the results of testing of the X3 and X4 positions reflected P2 and P3, respectively, or possibly other/mixed specificities. To test this, the reaction products of SplF with X3, X2, and X1 libraries (respective mixtures of all constituting sublibraries) were analyzed by LC-MS. A significant product peak corresponding to Abz-Tyr-Met-Leu-COOH had already been identified among the hydrolysis products of the Abz-Tyr-X3-X2-X1-Anb library, demonstrating that substrate preference determined using the X4 library corresponds primarily to P3 specificity of SplF. In other words, the design of the substrate itself, unintentionally ensured that X4 corresponded to the P3 position. Corresponding results were obtained when the X2 and X1 libraries were analyzed, indicating that the X3 and X2 preferences corresponded to the P2 and P1 specificities, respectively. The P1 specificity established using LSTS corresponded to the results obtained using ACC monitored selection, demonstrating that the cathepsin/caspase oriented design of the later libraries had no significant influence on the results of the P1 specificity assessment of SplF.

Concluding, we demonstrated that SplF is highly selective for leucine, and methionine at the P1 subsite. P2 is relatively unspecific, while aromatic residues except tryptophan are preferentially selected at P3. The P1’ subsite is relatively selective for serine, however, hydrophobic sidechain containing residues are also accepted (except for Trp).

### 2.4. Influence of Precise N-Terminal Trimming on the Activity of SplF Protease

A previous study demonstrated the influence of precise N-terminal trimming on the activity of SplB protease by a mechanism distinct to the canonical activation of chymotrypsin protease zymogens [23]. To evaluate if a mechanism similar to that described in SplB controls the activity of SplF, we compared the kinetics of synthetic substrate hydrolysis by protease variants, artificially extended at the N-terminus (His_6_-SplF and Met-Gly SplF-His_6_) to the recombinant protease having the N-terminus identical to the mature, wild-type SplF (SplF-His_6_). Michaelis constants (K_M_), characterizing the variants extended at the N-terminus, were lower compared to that characterizing the variant containing the N-terminus identical to the wild type mature protease, demonstrating increased (~1.5x) substrate affinity of the extended variants (Table 1). At the same time, the N-terminal extensions significantly decreased the rate of substrate hydrolysis (k_cat_). Both extended variants of SplF were characterized by more than five times lower k_cat_ compared to the mature, wild type N-terminus containing enzyme. Correspondingly, the catalytic efficiencies (k_cat_/K_M_) of both N-terminally extended variants were at least 3.7 times lower compared to the wild type N-terminus containing variant, demonstrating the role of exact N-terminal trimming in shaping the activity of the SplF protease. Both the decrease in K_M_, and especially the decrease in k_cat_ and k_cat_/K_M_ values, were more significant in the case of the short, methionine-glycine N-terminal extension compared to that containing six histidines.

### 2.5. Crystal Structure of SplF Protease Uncovers the Molecular Determinants of Substrate Specificity

To characterize the molecular determinants guiding SplF substrate specificity, we solved the x-ray structure of the protease. SplF crystallized in the P1 space group with four molecules in the unit cell. In the further description, values representative for all chains are reported for chain A while differences among molecules in the unit cell are addressed with reference to the relevant chains. The structure was solved by molecular replacement at a 1.7 Å resolution. Data collection and refinement statistics are summarized in Table 2. A major part of the molecule was well defined by its electron density save for residues 123–126 (all chains), residues 177–179 (chain C), and 173–182 (chain D). The overall structure of SplF had a chymotrypsin-like fold characteristic for the S1 serine protease family. The protease was composed of two perpendicular β-barrels (17–90, 112–189) connected by a stretched linker (91–111). The active site was located at the interface of the two barrels. The catalytic triad was not performed, but this was only because His39 was mutated to Ala in our construct to obtain efficient expression, while the backbone and Cβ atoms of all three catalytic residues in SplF were roughly in their canonical orientations in all chains. The oxyanion hole was well performed in two (A, B) of the four chains contained in the asymmetric unit, while the Pro153-Gly154 peptide bond did not support the canonical oxyanion hole in chain D, and the entire Gln152-Ser156 region had noncanonical orientation in chain C. This is likely because loop 2 (Lys176-Glu180) was disordered in chains C and D due to crystallographic packing, while it stabilized loop 1 (Ile150-Gly157) in chains A and B (Figure S2). Other loops that slightly differed in orientations among chains were located away from the substrate recognition cleft. The P1 substrate binding pocket was composed of loop 1, loop 2, and a β strand (Tyr172-Asn175) constituting a part of one of the β-barrels. The P1 subsite was lined by the mainchain atoms of the said residues, Cβ of Ser155, and the sidechains of Ile151, Ile171, and Ser181, which are exposed to the binding pocket. As such, the pocket is relatively hydrophobic, with only some polar accents, and does not contain any moieties capable of salt bridge formation. Such characteristics agree with the primary specificity of SplF.

Structural similarity searches using algorithms implemented in the DALI server [24] showed that among proteins of known structure, the SplD protease [15] was most closely structurally related to SplF. Superposition of SplF and SplD yielded low RMSD of 0.7 Å (for 199 equivalent Cα atoms), in agreement with high, 94% sequence similarity. Sequence homology among Spl proteases was generally high and ranged from 94% to 45% (Figure S3). In fact, all Spl proteases and even broader, all staphylococcal serine proteases were closely structurally related. The structures of SplA and SplB [13,14] overlapped with SplF with RMSDs of 1.5 Å (192) and 1.5 Å (193), respectively. Comparable values characterize the structures of SplF and further Spl proteases. The structures of V8 [25] protease and epidermolytic toxins A and D overlapped with SplF with RMSDs of 1.8 Å (190), 2.0 Å (187), and 1.7 Å (185), respectively, again documenting significant structural homology. Finally, the structure of trypsin, the type protease of the S1 family was characterized by RMSD of 2.2 Å (177) when overlaid with that of SplF. The close structural homologies between staphylococcal serine proteases allow comprehensive structure-specificity analysis.

## 3. Discussion

### 3.1. Substrate Specificity of SplF

The Spl proteases characterized to date in terms of substrate specificity fall into one of the four major types of activities, described to date in the S1 family. SplA is characterized by chymotrypsin-like specificity [13]. SplB exhibits glutamyl peptidase activity, although shifted toward glutamine instead of glutamic acid [14]. SplD has specificity comparable to elastase [15]. SplE, in turn, is characterized by a novel type of P1 substrate specificity, distinct from all previously characterized S1 family members and recognizes histidine, and to a lesser extent, glutamine at the P1 subsite [16]. SplC seems proteolytically inactive, or is possibly activated only by a very specific, and yet unknown substrate [17]. These prior results argue that Spl proteases have non-overlapping specificities at the P1 subsite. Here, we defined the substrate specificity of the last protease encoded in *spl* operon - SplF. Our results indicate that SplF specifically recognizes large, aliphatic sidechains of methionine and leucine at the P1 position. Such specificity complements that of the previously characterized Spl proteases, suggesting that *spl* operon encodes a unique proteolytic system in staphylococci. Interestingly, none of the known staphylococcal serine proteases demonstrated trypsin activity (lysine/arginine specificity), a common type of specificity, among family S1 proteases. In fact, none of the secreted staphylococcal proteases exhibited trypsin specificity. Additionally, unlike the specificity of most known S1 family proteases, the specificity of all tested Spl proteases exceeded beyond the P1 subsite. Altogether, this may suggest that proteases encoded in the *spl* operon physiologically target a specific subset of substrates only. As such, the role of *spl* operon proteases in staphylococcal physiology remains to be determined, the elucidation of which is a topic of recent vivid investigation [10,11].

The P1 substrate specificity of SplF is unique among S1 family proteases. Chymotrypsin also hydrolyzes substrates with leucine and methionine at P1, but additionally accepts aromatic sidechains, which is not the case for SplF. This unique specificity of the SplF protease is guided by the particular design of the substrate binding pocket, as shown in this study.

### 3.2. Presumed Interactions Guiding the Recognition of the Substrate at the Active Site of SplF

**Figure 3:**
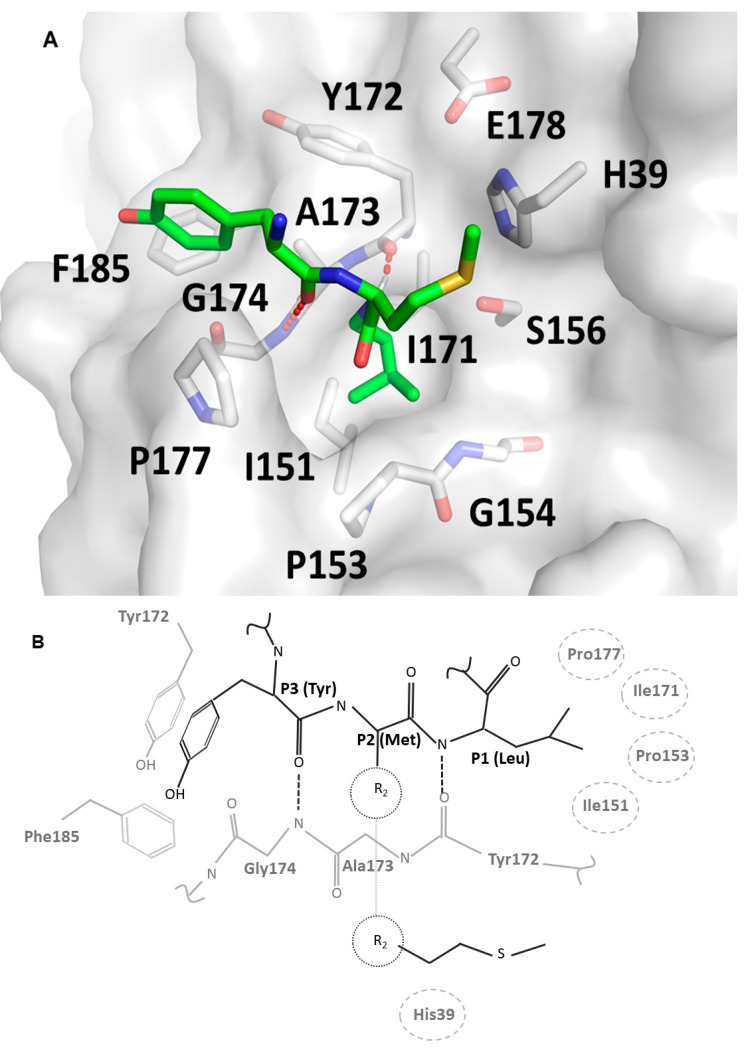
Model of consensus substrate recognition by SplF protease. (**A**) The overall orientation of the substrate (green) backbone was modeled based on the crystal structure of substrate mimetic inhibitors in complex with trypsin. The positions of the sidechains were adjusted by energy minimization. SplF (gray) residues involved in active site formation are highlighted in the stick model and labeled. (**B**) Schematic representation of substrate (black line)–enzyme (gray line) interaction. Hydrogen bonds are shown as dashed lines. Ring stacking is indicated. The enzyme residues involved in hydrophobic interaction are indicated by dashed ovals.

Substrate specificity profiling and structural data provided in this study, together with homology modeling based on the crystal structure of trypsin in complex with substrate mimetic inhibitors, allowed us to propose a model of substrate binding at the active site of the SplF protease (Figure 3). The backbone of the substrate docks at Tyr172-Asn175 β-strand, forming two characteristic hydrogen bonds (at Tyr172 and Gly174) resembling an antiparallel β sheet. The model proposes, that the P3 specificity is dictated by π–π and oxygen lone pair π interactions of P3 tyrosine sidechain at a shallow pocket composed by sidechains of Tyr172 and Phe185 of the enzyme. The P2 sidechain would point away from the enzyme, providing only weak hydrophobic interaction of its Cβ at the face of the active site histidine imidazole ring, which would explain broad specificity at the P2 subsite. The P1 sidechain, in turn, is buried in a deep, hydrophobic pocket lined with the sidechains of Ile151, Pro153, Ile171, and Pro177, which explains the propensity of the enzyme for large, hydrophobic sidechains of methionine and leucine at the P1 position. Smaller hydrophobic sidechains are not able to fill the P1 pocket entirely, while bulky aromatic sidechains are not sterically compatible.

### 3.3. Determinants of SplF Substrate Specificity

**Figure 4:**
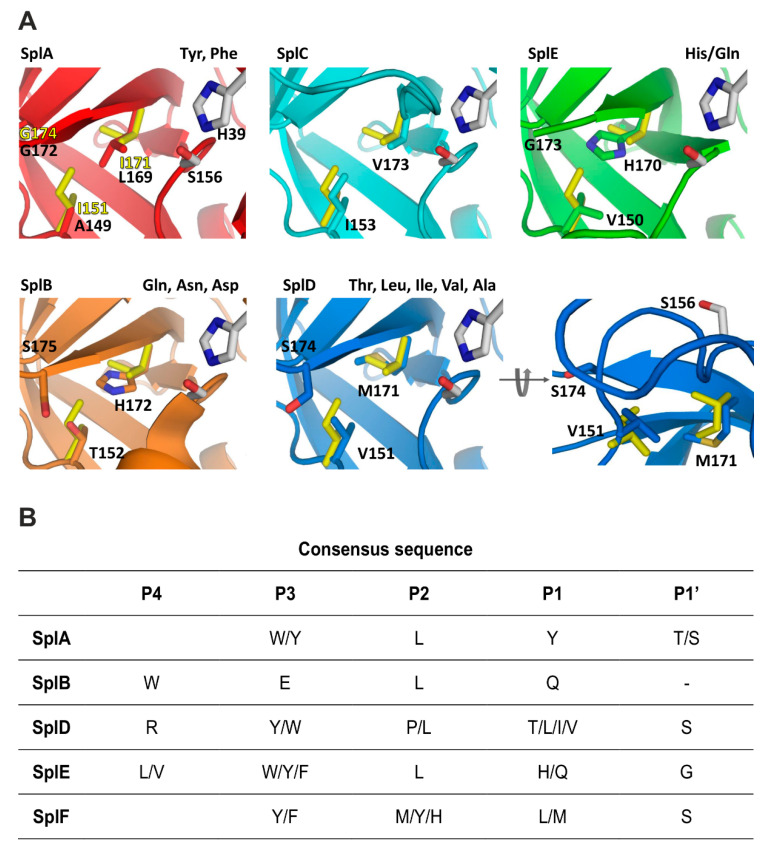
S1 specificity pockets of Spl proteases in the context of their substrate specificities. (**A**) The residues defining the S1 pocket of SplF (PDB 6SF7, yellow) are overlaid on relevant pockets of other proteases of the *spl* operon: SplA (PDB 2W7S, red) [13], SplB (PDB 2VID, orange) [14], SplC (PDB 2AS9, cyan) [17] and SplD (PDB 4INK, blue) [15] and SplE (PDB 5MM8, green) [16]. Catalytic triad residues are highlighted grey. Residues exposed at the S1 pocket and thus of direct significance in specificity determination are shown in stick model and colored according to the compared proteases. See Figure S4 for broader overview of the binding cleft in compared enzymes. (**B**) Consensus sequences recognized and cleaved by Spl proteases characterized to date.

Given the differences in P1 substrate specificity and close structural relatedness of Spl proteases and, more generally, all S1 family serine proteases, it was interesting to revisit the structural determinants within this family in light of the accumulated data (Figure 4, Figure S4). S1 family proteases are built on a common scaffold, and the P1 substrate specificity is determined *via* differences in the composition of sidechains exposed at the binding pocket. Although simple, this mechanism is sufficient to determine a large variety of specificities, where different P1 pocket designs allow it to accommodate only sidechains characterized by particular physicochemical properties. Thereby, a relatively straightforward analysis of the S1 pocket composition provides information on the determinants of enzyme specificity. Truly, seminal works on trypsin, chymotrypsin, and elastase have established a charge/polarity, and a steric constraints guided model to explain the P1 specificities of the above enzymes [26]. This model also roughly explains the specificities of other S1 family proteases, since their S1 binding pockets are all based on the same design and differ primarily at several residues exposed to the S1 pocket, thus modifying its properties.

Bovine chymotrypsin selects substrates containing Tyr, Phe, and Leu at the P1 subsite and additionally accepts those with Trp, Met, Asn, and His. The specificity of SplA is more restricted, limited almost exclusively to Tyr and Phe. Accordingly, the S1 binding site of chymotrypsin is a bulky cavity, where a number of interactions with the substrate are mediated by water molecules. In SplA, the waters are replaced with the sidechains of Asn181 (Gly226 in chymotrypsin), Leu169 (Val213), and Ser178. Such a decrease in plasticity manifests in a more restricted substrate preference (Figure 4). SplF selects large aliphatic residues, but excludes aromatic sidechains. This is because in SplF, Ile151 restricts the size of the pocket compared to SplA (corresponding Ala149). Additionally, Asn181, which provides the carbonyl–π interaction with aromatic P1 residue of a substrate in SplA, is absent in SplF (corresponding Gly184). SplD prefers P1 residues with short sidechains. This is because the S1 pocket of SplD is even more restricted compared to SplF and SplA, which is due to the sidechain of Ser174 located at the pocket entrance, where glycine is present at the corresponding position in both latter proteases. The specificity of SplD is roughly comparable to that of elastase and so are the S1 binding pockets (Val216 is found in elastase at the position equivalent to Ser174 in SplD).

The general preference of SplB, SplE, and V8 proteases for polar sidechains is determined by interaction contributed by imidazole ring exposed at the bottom of the P1 pocket and further modulated by the surrounding residues. SplE selects substrates containing histidine (and to a lesser extent glutamine) because the hydrophobic walls of the pocket (Val150, Gly173) provide favorable interactions with the aromatic ring. SplB, in turn, prefers asparagine, glutamine, and aspartic acid (and to a lesser extent histidine) because of additional interactions provided by corresponding Thr152 and Ser175. It has been demonstrated that converting the SplB P1 specificity pocket to that of SplE (Thr152Val and Ser175Gly) converts the primary specificity of the former protease toward that of the latter. The primary preference of V8 protease is toward glutamic acid. Compared to SplB (primary specificity for glutamine), V8 protease specificity is determined by a positively charged moiety (N terminal amine) present exclusively at the binding pocket of V8, due to its unique design compared to all the other chymotrypsin family proteases.

The crystal structure of SplC has demonstrated that the P1 binding pocket is strongly deformed compared to a canonical fold and the protein has never been demonstrated to exhibit hydrolytic activity. It remains unknown if SplC is activated by structural rearrangement at some unknown conditions or if its function is expressed by means other than proteolysis.

### 3.4. N-Terminal Processing Controls the Activity of SplF Protease

Expression of proteolytic activity requires tight control to minimize unwanted hydrolysis of vital cell components. Two major mechanisms include the production of proteases in the form of inactive zymogens activated only at the target sites, and expression of proteinaceous inhibitors.

The majority of serine proteases of the S1 family are produced as inactive zymogens. Only the removal of an N-terminal extension by proteolytic processing allows refolding of the enzyme’s active site liberating the proteolytic activity [27,28]. Such a mechanism protects the secreting cell cytoplasm against unwanted proteolysis by misdirected secretory enzymes. The imperfection of secretory pathways in staphylococci, and the requirement for protection of cytoplasmic proteins against secretory proteases, is best exemplified by staphopains. Although staphopains are directed for secretion and produced in a zymogen form, these cysteine proteases are still accompanied by dedicated cytoplasmic inhibitors of nanomolar affinity [29]. Only such multilevel control ensures the protection of intracellular components from unwanted proteolysis.

Spl proteases are secreted in an already active, mature form, and intracellular serine protease inhibitors have not been documented to date in S. *aureus*. Nevertheless, the activity of Spls is still under strict functional control. It has been demonstrated that a secretion signal in the SplB protease fulfills a double role, directing the enzyme to the extracellular compartment, and limiting its activity if misdirected to the cytoplasm [23]. Here, we extended the above finding on SplF, demonstrating that, identically to SplB, the N-terminal extension limits the specific activity of the protease roughly tenfold. This phenomenon is not related to any specific properties of the signal peptide. Instead, any modifications of the native N-terminus result in the decreased proteolytic activity of Spls. This is because the N-terminal primary amine is necessary for the full folding of the enzyme (the molecular mechanism is distinct from that described for canonical chymotrypsin-like protease zymogens and likely related to changes in the overall tumbling dynamics [23]). Such a mechanism allows unrestricted evolution of the secretion signal function without affecting the activity control. Since all Spl proteases are similar in their structures, it seems that combining the roles of a secretion signal, and an inhibitory profragment is a universal feature in these enzymes.

The signal peptides are removed by secretion machinery during translocation, releasing a mature N-terminus. Thus, Spl proteases are activated directly during secretion, while the population misdirected to the cytoplasm contains a signal peptide, and thus exhibits only a limited activity. Such a decrease in activity is sufficient to protect the cytoplasm against minute quantities of misdirected enzymes. The conclusion is supported by the fact that we were able to overexpress N-terminal modified variants of SplF in *E. coli* only at low level (His_6_-SplF, Met-Gly-SplF-His_6_), while high level expression required total inactivation of proteolytic activity (Gly-Ser-SplF [His39Ala]). Given such a mechanism, the decreased activity of N-terminally elongated forms of Spl proteases seems a physiologically relevant phenomenon.

### 3.5. Perspective

With the current study, the staphylococcal proteases of the *spl* operon have now been thoroughly characterized in terms of structure and biochemistry. The role of the signal peptide has been documented as a novel mechanism in controlling enzyme activity at undesired sites. Concert expression and complementary primary substrate specificities suggest concomitant action of Spl proteases, while extended specificities suggest a more specific role than simple nutrient acquisition. In this context, it is most interesting to determine if SplC is truly a protease active against a very limited set of substrates, or has yet another role, not related to proteolysis. Operons containing more than six Spl like proteases have been identified in staphylococci other than *S. aureus*. It is interesting to determine if the specificities of those additional proteases are further complementary, especially since Spls have also found utility in biotechnology (fusion tag removal) due to their restricted specificity, and additional enzymes of new specificities might complement those already in use. However, currently, the most fascinating studies aim at elucidating the physiological function of Spl proteases. The seminal report suggests the involvement of SplC in endocarditis [7]. More recent studies document the likely role of Spls in the etiology of allergic asthma, and SplA in disseminated pneumonia [10,11]. Certain convincing mechanisms were also suggested. However, further studies are clearly needed to fully elucidate the role of Spl proteases in staphylococcal borne disease. This is facilitated, among others, by the knowledge and tools (sensitive substrates) developed in our biochemical studies.

## 4. Materials and Methods

### 4.1. Expression and Purification of SplF Protease Variants

All tested variants of SplF (UniProt number: Q2FXC8) protease were expressed in *Escherichia coli* as an N-terminal GST fusion or N-/C-terminal His_6_ tag fusions. Gene encoding mature SplF(37-230) was PCR amplified from genomic DNA of *S. aureus* strain 8325-4 (Table S1) and cloned into BamHI/XhoI sites of pGEX-5T plasmid [18]. The His39Ala mutant was obtained by site-directed mutagenesis. Constructs encoding His_6_-SplF and Met-Gly-SplF-His_6_ were prepared in pETDuet-1 using NcoI/BamHI and NcoI/HindIII sites, respectively. SplF-His_6_ was cloned at NcoI/HindIII sites of pET-20b(+) in frame with PelB secretion signal.

Gly-Ser-SplF(His39Ala) was expressed in BL21 pLysS(DE3) strain. Bacteria were cultured in LB supplemented with 100 mg/L ampicillin, at 37 °C until OD_600_ reached 0.6 and then at room temperature (RT) until the OD_600_ reached 1. The protein expression was induced by the addition of 1 mM IPTG and carried out for 4 h at RT. Bacteria were harvested by centrifugation, pellets were washed with ice-cold PBS, and lysed by sonication. Cell debris was removed by high speed centrifugation. Recombinant GST fusion protein was recovered on glutathione sepharose (GE Healthcare, Uppsala, Sweden). After elution, the tag was separated with thrombin (Sigma-Aldrich, Saint Louis, Missouri, USA) and GlySer-SplF (His39Ala) was purified by ion exchange chromatography (MonoS; GE Healthcare, Uppsala, Sweden) in 30 mM sodium acetate, pH 5.0. The protein was further purified, and the buffer exchanged using gel filtration (Superdex s75; GE Healthcare, Uppsala, Sweden) in 5 mM Tris-HCl, 50 mM NaCl, pH 8.0. The quality of obtained preparations was verified using SDS-PAGE, and only preparations of 95% and higher purity was used for further analysis.

All remaining constructs were expressed in the BL21(DE3) strain. Bacteria were cultured at 37 °C until OD_600_ reached 0.6 and then at 22 °C until OD_600_ reached 1. Protein expression was induced with 1 mM IPTG and continued for 6 h. For His_6_-SplF and Met-Gly-SplF-His_6_, the bacteria were suspended in PBS and lysed by sonication. For SplF-His_6_, the protein was salted out of the cell culture supernatant with ammonium sulfate at a final concentration of 85% of the saturated solution and recovered by centrifugation. Residual ammonium sulfate was removed by dialysis. In all cases, the proteins were recovered on chelating sepharose (GE Healthcare, Uppsala, Sweden) and further purified by ion exchange chromatography (MonoQ; GE Healthcare, Uppsala, Sweden) in 50 mM Tris-HCl, pH 7.5. All preparations were formulated in PBS by dialysis, the purity was evaluated by SDS-PAGE and the proteolytic activity by zymography using β-casein as a substrate.

### 4.2. Zymographic Analysis

Zymographic analysis of protein samples was performed as described earlier in Popowicz et al. [17]. In brief, samples were mixed with Laemmli loading buffer (4×, without β-mercaptoethanol) and incubated for 10 min at room temperature. Next, the samples were loaded on 12% (*w*/*v*) polyacrylamide gel containing 0.1% (*w*/*v*) bovine β-casein (Sigma) and 0.04% SDS and electrophoresed. The proteins were then renatured by incubation in 2.5% (*v*/*v*) Triton X-100 in water (2 × 30 min) at room temperature with agitation. Gels were developed in 100 mM Tris-HCl (pH 7.8), 100 mM NaCl, 5 mM EDTA for 3 h at 37 °C and stained with Amido black. Zones of hydrolysis were visualized by destaining in 10% (*v*/*v*) acetic acid solution.

### 4.3. Characterization of SplF Substrate Specificity at P1 Position

Libraries of fluorogenic substrates: Ac-Ala-Arg-Leu-P1-ACC and Ac-Leu-Arg-Ser-P1-ACC were synthesized as described previously [20,21]. Each library contained 20 sub-libraries each having a fixed proteinogenic amino acid or norleucine at the P1 position. Libraries (200 µM) were screened with SplF (4.67 µM) in PBS at 37 °C by monitoring fluorescence release (Ex = 355 nm, Em = 460 nm). The slope of the linear fragment of hydrolysis curve was used as a measure of protease activity against each sublibrary.

### 4.4. Synthesis of Peptide Libraries

ABZ-X4-X3-X2-X1-ANB-NH2 libraries were synthesized manually on solid support using Fmoc/tBu chemistry and the mixing-portioning method [30]. A total of 23.6 g of the resin (TentaGel S RAM resin, substitution level of 0.25 meq/g; Rapp Polymere, Tübingen, Germany) was used. The synthesis was initiated by deprotection of the amino groups of the resin with 20% piperidine in 1-methyl-2-pyrrolidone (NMP), then the resin-bound amino groups were acylated with 5-amino-2-nitrobenzoic acid using the N,N,N’,N’-tetramethyl-O-(benzotriazol-1-yl)uranium tetrafluoroborate (TBTU)/4-dimethylaminopyridine (DMAP). The resin was washed twice with N-methylmorpholine. Next, two equivalents of ANB and two equivalents of TBTU/DMAP were dissolved in DMF and added to the resin. Upon 30 s, four equivalents of N,N-diisopropylethylamine (DIPEA) were added. The whole mixture was stirred for 3 h. To assure complete resin substitution, the procedure was repeated three times. The first Fmoc-protected amino acid was conjugated to ANB using a POCl_3_/pyridine system [31] and deprotected. All subsequent equimolar mixtures of Fmoc-protected amino acids were coupled using N,N’-diisopropylocarbodiimide (DIPCI)/1-hydroxybenzotriazole (HOBt) system in threefold excess. Finally, the Boc protected 2-aminobenzoic acid was introduced as the N-terminal group.

The obtained peptides were cleaved from the resin using a trifluoroacetic acid (TFA)/phenol/triisopropulsiane/H_2_O mixture (88:5:2:5, *v*/*v*) [32].

### 4.5. MS Analysis

Libraries and peptides after synthesis, and cleavage products were analyzed using RP-HPLC (Jasco, Tokyo, Japan) equipped with Supelco Wide Pore C8 column (8 × 250 mm) and ultraviolet-visible (UV-VIS, 226 nm) and fluorescent (excitation 320 nm, emission 450 nm) detection. A linear gradient from 10 to 90% B within 40 min was applied (A: 0.1% TFA in water; B: 80% acetonitrile in A). Mass spectrometry analysis was performed using a Biflex III MALDI TOF mass spectrometer (Bruker, Karlsruhe, Germany) and α-cyano-4-hydroxycynnamic acid (CCA) or 2,5-dihydroxybenzoic acid (DHB) as a matrix.

### 4.6. Substrate Specificity Profiling Using Libraries of Synthetic Tetrapeptide Substrates (LSTS)

Substrate specificity profiling of SplF was performed using Libraries of Synthetic Tetrapepdide Substrates (LSTS) essentially as described previously [33]. In brief, a library of a general structure Abz-X4-X3-X2-X1-Anb-NH2 was synthesized on a solid support as described above, where Anb (5-amino-2-nitrobenzoic acid) quenches the fluorescence of Abz (aminobenzoic acid). The initial library contained 19 sublibraries, each of which contained a defined proteiongenic amino acid at the X4 position, and an equimolar mixture of those residues at X3, X2, and X1. Each sublibrary (50 ug/mL) was probed with SplF (6.9 × 10^−4^ to 2.76 × 10^−5^ M for the following subsites; varied to obtain detectable/unsaturated readings) in 180 µL of 20 mM Tris-HCl (pH 8.0) at 37 °C while monitoring the released fluorescence (Ex = 320 nm, Em = 410 nm) using an Omega plate reader (BMG, Labtech, Ortenberg, Germany). For each subsite, the results were normalized at the most active sublibrary. The maximal theoretical diversity of any of the tested sublibraries was accommodated within such test design with significant redundancy. The X4 position was fixed with the most efficiently recognized residue and a library was synthesized containing 19 sublibraries, each of which had a fixed residue at the X3 position. LSTS were deconvoluted in the above manner until all the positions of the substrate were fixed with optimally recognized residues. The site of hydrolysis was determined by LC-MS.

### 4.7. Enzyme Kinetics

Kinetic parameters were determined as described previously [33] in PBS at 37 °C at a SplF concentration of 2.76 × 10^-5^ M. All measurements were performed in technical triplicates and systematic error expressed as a standard deviation never exceeded 20%. K_M_ and V_max_ were determined by fitting the Michaelis–Menten equation using the GraphPad Prism Enzyme Kinetic software (GraphPad Software, San Diego, CA, USA). The specificity constant (k_cat_/K_M_) was calculated from k_cat_ and K_M_ values.

### 4.8. Crystallization and Structure Determination

Gly-Ser-SplF(His39Ala) was concentrated to 15 mg/mL and used for crystallization screening in a sitting-drop vapor-diffusion setup. Two μL drops and 1:1 ratio of protein to reservoir were used with a reservoir volume of 150 μL. Several initial crystallization conditions were identified and further optimized. Single crystals used for data collection were obtained in an optimized condition containing 32.5% (*v*/*v*) PEG 400 and 0.3 M ammonium sulfate in 0.1 M sodium citrate pH 3.5. Crystals were flash-cooled in liquid nitrogen using 30% glycerol in mother liquor as a cryoprotectant. Diffraction data were collected on an in-house x-ray source (Rigaku Micromax-007 HF equipped with R-AXIS IV++ imaging plate detector, Tokyo, Japan). Data were analyzed using programs contained in the CCP4 program suite [34]. Images were processed in iMosflm and the data were scaled with Scala [35]. Molecular replacement was performed in Phaser [36] with alanine search model constructed based on the structure of SplB (PDB: 2VID, chain A) [14]. Model building and refinement were performed using COOT [34] and Refmac5 [37], respectively. Data collection and refinement statistics are summarized in Table 2. The refined model and experimental data are available at the Protein Data Bank (6SF7).

### 4.9. Molecular Modeling

The structure of one of the substrate peptides (YML) was created in Discovery Studio Visualizer (Dassault Systèmes BIOVIA, Vélizy-Villacoublay, France) and subsequently optimized in VEGA ZZ molecular modeling software. The peptide was then docked with AutoDock Vina [38] to the model of the SplF created in the SWISS-MODEL [39] server (to account for the orientation of the His39 sidechain, which was mutated to Ala in the crystal structure reported in this study). The orientation of the peptide was selected, which resembled that expected for the substrate based on the structures of substrate mimetic inhibitors in complex with trypsin (ex. PDB code 4XOJ).

## Figures and Tables

**Table 1:** Kinetics of ABZ-Tyr-Met-Leu-Ser-ANB-NH_2_ hydrolysis by different SplF protease variants.

SplF Variant	Modifications	K_M_ [µM]	k_cat_ [s^−1^]	k_cat_/K_M_ [M^−1^s^−1^]
His_6_-SplF	N-terminus: His_6_	21.4 (±1.5)	0.07 (±0.02)	3271 (±1164)
Met-Gly-SplF-His_6_	N-terminus: Met-Gly * C-terminus: His_6_	18.2 (±3.9)	0.015 (±0.001)	824 (±232)
SplF-His_6_	C-terminus: His_6_	30.8 (±2.9)	0.40 (±0.03)	12,987 (±2197)
Gly-Ser-SplF(His39Ala)	N-terminus: Gly-Ser ^$^	nr ^#^	nr ^#^	nr ^#^

Standard deviation (SD) values are provided in parentheses. * Met-Gly in the construct arises as an artifact of cloning (remnant of the restriction site). ^#^ Proteolytically inactive catalytic triad histidine mutant (no expression of wild type was obtained). ^$^ Gly-Ser remain as a part of protease recognition/hydrolysis site.

**Table 2:** Crystallographic data collection and refinement statistics.

Wavelength (Å)	1.54
Resolution range (Å)	30.00–1.70 (1.79–1.70)
Space group	P 1
Unit cell	56.00 58.58 62.30 104.57 95.31 90.6
Total reflections	283,284 (39,749)
Unique reflections	77,687 (10,882)
Multiplicity	3.6 (3.7)
Completeness (%)	92.6 (88.8)
Mean I/sigma(I)	13.9 (6.4)
Wilson B-factor	20.25
R-merge	0.047 (0.110)
R-meas	0.055 (0.129)
R-work	0.1674
R-free	0.2078
Number of non-hydrogen atoms	6562
Macromolecules	5996
Water	451
Protein residues	755
RMS (bonds)	0.168
RMS (angles)	2.050
Ramachandran favored (%)	96.6
Ramachandran outliers (%)	0.00
Clashscore	2.39
Average B-factor	27.70
Macromolecules	26.60
Solvent	35.45

Data for the highest resolution shell are provided in parentheses.

## Data Availability

Three dimensional atomic coordinates and structure factors of SplF have been deposited in the Protein Data Bank, www.pdb.org (PDB ID code 6SF7).

## Supplementary Materials

The following are available online at https://www.mdpi.com/1422-0067/22/4/2220/s1, Figure S1: Deconvolution scheme of a fluorogenic tetrapeptide library (LSTS) against SplF protease, Figure S2: Overall fold of SplF and comparisons of chains contained in the asymmetric unit, Figure S3: Sequence alignment of Spl proteases, Figure S4: Comparison of substrate binding clefts of Spl proteases, Table S1: Primers used to generate expression constructs.
