# Supplementary material for: Structural Determinants of Substrate Specificity of SplF Protease from Staphylococcus aureus
Source: Int J Mol Sci. 2021 Feb 23;22(4):2220. doi: 10.3390/ijms22042220 (PMC7926377; doi:10.3390/ijms22042220)
Supplement: Supplementary file 1 [file ijms-22-02220-s001.pdf]

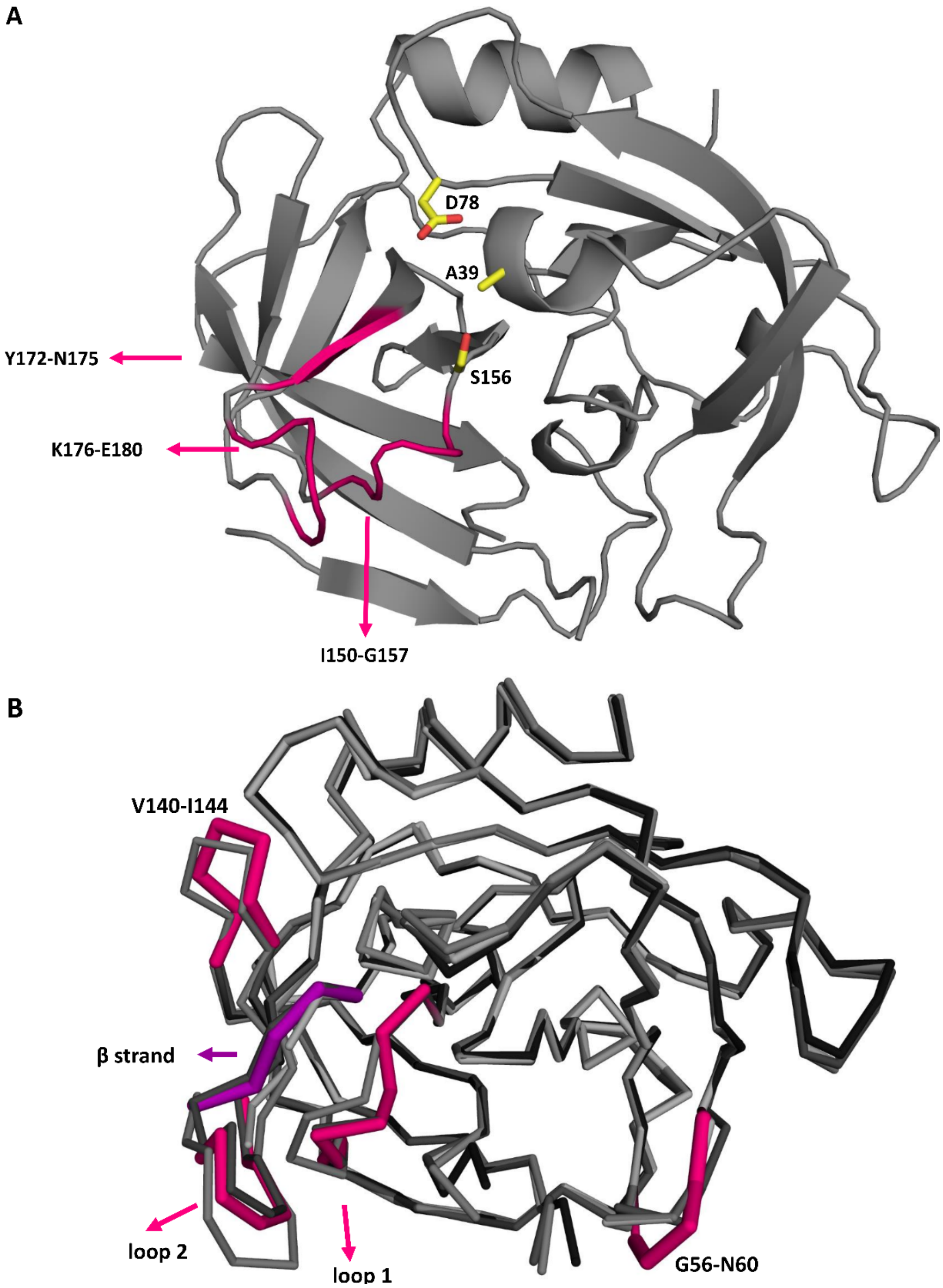

Figure S2. Overall fold of SplF (A) and comparisons of chains contained in the asymmetric unit (B). (A) Overall fold of SplF protease. Primary features described in the text are highlighted. (B) Superposition of chains contained in the unit cell (grey tones). Major differences among chains are observed in loops 1 and 2 constituting the binding pocket and addi-tionally in fragments V140-I144 and G56-N60 (highlighted in colour).

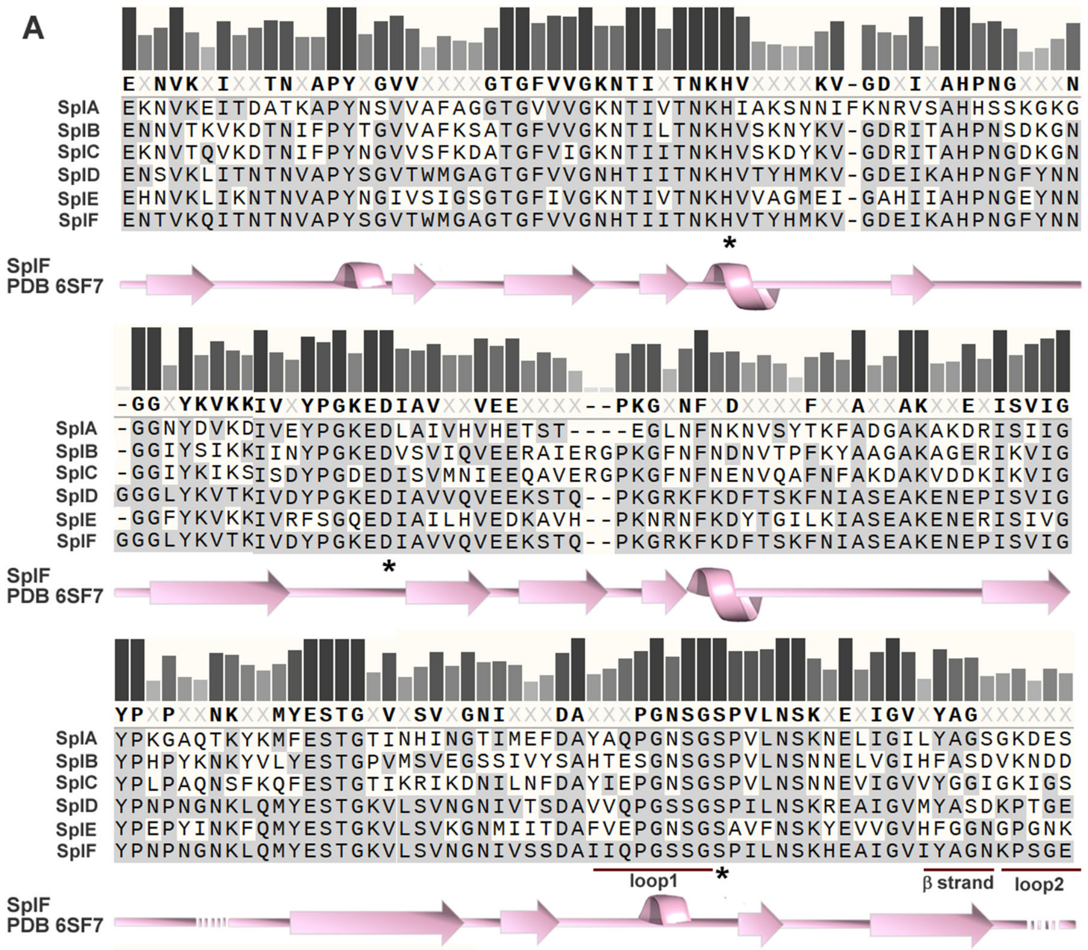

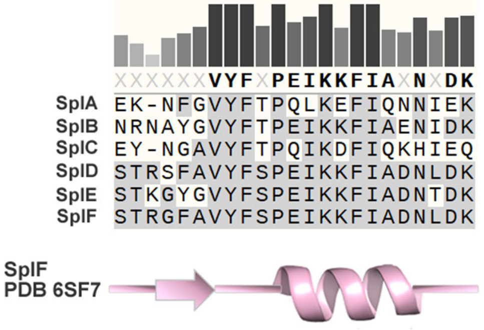

B

SplA	100%					
SplB	48%	100%				
SplC	50%	63%	100%			
SplD	44%	54%	50%	100%		
SplE	43%	56%	50%	67%	100%	
SplF	45%	54%	51%	94%	68%	100%
Spl	SplA	SplB	SplC	SplD	SplE	SplF

Figure S3. Sequence alignment of Spl proteases.

(A) The sequences of Spl proteases were alignment to the sequence of SplF. Secondary structure elements (SplF) are shown below the sequence. The restudies of the catalytic triad (asterisk) and residues involved in S1 pocket formation (loop1, loop2 and β strand; underlined) are highlighted.

(B) Amino acid sequence identities calculated using paired alignments are shown for particu-lar Spl proteases.

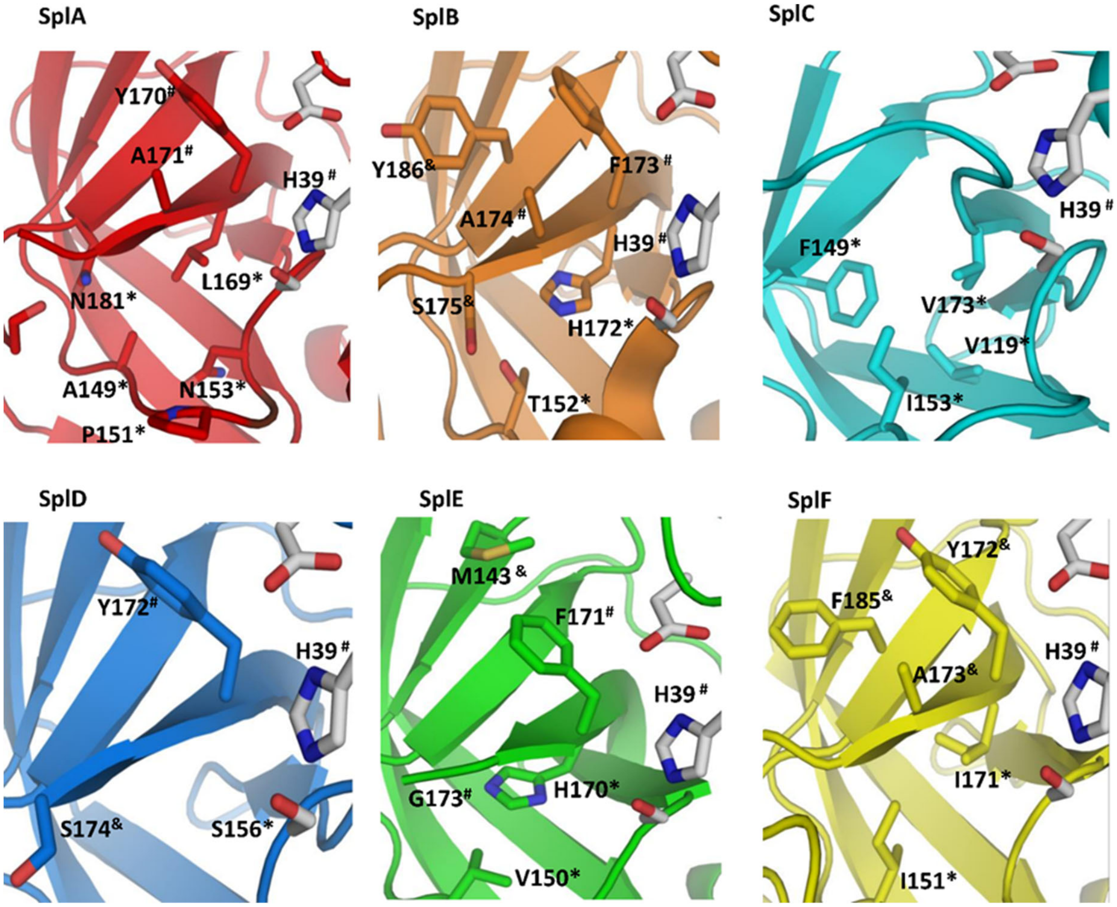

Figure S4. Comparison of substrate binding clefts of Spl proteases.

The residues defining the S1 (*), S2 (#), and S3 (&) pockets of Spl proteases are shown in stick model: SplA (PDB 2W7S, red) [13], SplB (PDB 2VID, orange) [14], SplC (PDB 2AS9, cyan) [17] and SplD (PDB 4INK, blue) [15], SplE (PDB 5MM8, green) [16] and SplF (PDB 6SF7, yellow). Residues constituting the catalytic triad are highlighted grey.
